# Author Correction: Minimal system for assembly of SARS-CoV-2 virus like particles

**DOI:** 10.1038/s41598-021-88846-9

**Published:** 2021-04-26

**Authors:** Heather Swann, Abhimanyu Sharma, Benjamin Preece, Abby Peterson, Crystal Eldredge, David M. Belnap, Michael Vershinin, Saveez Saffarian

**Affiliations:** 1 Center for Cell and Genome Science, University of Utah, Salt Lake City, USA; 2 Department of Physics and Astronomy, University of Utah, Salt Lake City, USA; 3 School of Biological Sciences, University of Utah, Salt Lake City, USA; 4 Department of Biochemistry, University of Utah, Salt Lake City, USA

Correction to: *Scientific Reports* 10.1038/s41598-020-78656-w, published online 14 December 2020

The original version of this Article contained an error in the spelling of the author Crystal Eldredge which was incorrectly given as Crystal Eldridge. The original Article and accompanying Supplementary Information file have been corrected.

